# Interaction of OsCSN2 with OsCULs Under Red and Far-Red Light Regulates Stem and Coleoptile Growth in Rice

**DOI:** 10.3390/plants15010028

**Published:** 2025-12-21

**Authors:** Le Yin, Hua Zeng, Xinyue Jia, Zizhu Zhao, Zihao Wang, Elshan Musazade, Yanxi Liu, Miao Xu, Jingmei Lu, Liquan Guo, Ming Wu

**Affiliations:** 1 College of Life Sciences, Jilin Agricultural University, Changchun 130118, China; lyjiau@126.com (L.Y.); elshan.musazade1@gmail.com (E.M.);; 2 School of Food and Pharmaceutical Engineering, Wuzhou University, Wuzhou 543002, China; 3 Key Laboratory of Soybean Molecular Design Breeding, Northeast Institute of Geography and Agroecology, Chinese Academy of Sciences, Changchun 130102, China; 4 School of Life Sciences, Northeast Normal University, Changchun 130024, China

**Keywords:** OsCSN2, CRL-mediated ubiquitination, protein–protein interactions, red light, far-red light

## Abstract

CSN2, a highly conserved subunit of the COP9 signalosome (CSN), serves as the primary binding site for Cullin in the CSN complex. This interaction, dependent on lysine residues, positions CSN2 as a key player in approximately 20% of CRL-mediated ubiquitination reactions, a critical regulatory pathway for growth, development, and cellular processes in eukaryotes. While the role of CSN2 in human cells has been partially characterized, its function in rice (OsCSN2) remains poorly understood. Building on our previous findings regarding OsCSN2 function under natural light, this study investigates its regulatory mechanisms in rice seedlings under red and far-red light conditions. We demonstrate that under natural light, OsCSN2 mainly affects rice GA homeostasis by regulating the expression of SLR1 and influences rice photomorphogenesis by regulating the expression of the COP1-HY5 complex, thereby controlling rice growth through two pathways. Unlike under natural light, under red light, OsCSN2 promotes the expression of OsGID1, enhances the interaction between OsGID1 and OsSLR1, and promotes GA accumulation and OsPIL14 expression, leading to rice stem growth and inhibition of coleoptile elongation. Under far-red light, OsCSN2 mainly promotes the expression of OsCOP1, increasing the formation of the COP1-HY5 complex, which inhibits photomorphogenesis and coleoptile elongation. Lysine site mutations in OsCSN2 affect the interaction between the OsCSN complex and CRLs, regulating CRL-mediated ubiquitination reactions, promoting the ubiquitin-mediated degradation of OsSLR1 and OsCOP1, and thus promoting rice growth. These findings not only elucidate the functional roles of OsCSN2 in rice growth regulation but also provide valuable genetic resources for breeding rice varieties with enhanced agronomic traits.

## 1. Introduction

The COP9 signalosome (CSN) is a highly conserved heteromeric protein complex in eukaryotes that plays a crucial role in development and signal transduction. This complex consists of eight subunits, designated CSN1-CSN8, in descending order of molecular weight [1]. As a key regulator of Cullin-RING ubiquitin ligases (CRLs), the CSN complex inactivates CRLs by removing the small ubiquitin-like modifier NEDD8 (neural precursor cell-expressed developmentally downregulated 8), thereby participating in approximately 20% of Cullin-mediated ubiquitination reactions [2]. Structurally, CSN contains two primary domains (PCI and MPN): one that facilitates substrate receptor module (SRM) replacement to regulate CRL target specificity and another that accelerates complex formation by bridging CRLs through inositol hexakisphosphate (IP6) linkage [3].

CSN regulates various developmental processes in plants by interacting with multiple CRL complexes. In *Arabidopsis*, the phenotypes of different CSN subunit deletion mutants show distinct variations [4]. *csn* mutants exhibit a constitutive photomorphogenic growth pattern in the dark, with gene expression profiles resembling those under high-light conditions. CSN also modulates hormonal signaling pathways within plants. For instance, CSN directly interacts with SCF^TIR1^. In the absence of CSN, the efficiency of SCF^TIR1^-mediated degradation of AUX/IAA substrates is markedly reduced, resulting in a diminished auxin response [5]. Additionally, CSN interacts with SCF^COI1^, thus playing a role in jasmonic acid-mediated plant defense responses [6]. In the gibberellin (GA) pathway, CSN deficiency leads to the accumulation of the SCF^SLY1^ substrate, RGA, thereby affecting seed germination and stem elongation [7].

CSN2 is one of the most conserved subunits within the COP9 signalosome, with a C-terminal PCI domain that mediates interactions with the other subunits [8]. CSN2 binds to Cullins in a conserved manner, thereby modulating the activity of various CRL complexes [9]. It serves as the principal binding site for CRL4 in the CSN complex [10]. Inositol polyphosphate metabolites (IP6 and IP7) and their metabolic enzymes (IP5K and IP6K) have been shown to facilitate the assembly and stimulus-dependent dissociation of the CRL-CSN complex [3]. IP6 significantly enhanced the binding affinity between CRL4A and CSN, particularly by strengthening its interaction with CSN2. Even at sub-physiological concentrations, IP6 reduces the dissociation constant between CSN2 and CUL4A by 30-fold [11]. This effect is attributed to IP6 interacting with a highly basic region of CSN2, engaging seven lysine residues that are critical for its binding to CRLs [12].

Different monochromatic lights can influence plant growth and quality by modulating photosynthesis, photomorphogenesis, and secondary metabolism [13,14,15]. Plant responses under red light are mainly related to phytochromes [16,17]. Phytochromes interact with phytochrome-interacting factors (PIFs) to respond to red/far-red light signaling, regulating downstream light-mediated target gene transcription and controlling plant photomorphogenesis [18,19]. Phytochrome B (phyB) is the main receptor for red light in plants, mediating photomorphogenesis, thermomorphogenesis, and shade avoidance responses through interaction with PIFs [20,21]. Phytochrome A (phyA) is the only receptor in plants that senses and responds to far-red light signals, responsible for perceiving and mediating far-red light-related signaling pathways [22,23].

In light-signaling pathways, Constitutive Photomorphogenic 1 (COP1) acts as a critical negative regulator of photomorphogenesis. COP1-deficient mutants exhibit constitutive photomorphogenic development even in the dark [23]. COP1 contains three conserved domains: the N-terminal RING-finger domain, which interacts with CRLs; the C-terminal WD40 repeat domain, which binds to phyA or other substrates; and the central coiled-coil domain, which facilitates homodimerization or heterodimerization with other proteins [24,25]. HY5 is a core positive regulator at the terminal point of photomorphogenic pathways, and its abundance correlates directly with the extent of photomorphogenesis [26]. HY5 expression is upregulated in light and targeted for proteasomal degradation in the dark [27]. COP1 binds to HY5 within the nucleus and specifically ubiquitinates HY5 *in vitro*. Additionally, the CSN complex regulates the nuclear-cytoplasmic distribution of COP1. In *csn* mutants, the nuclear accumulation of COP1 was abolished, indicating that COP1 nuclear localization in the dark is CSN-dependent. Within the nucleus, COP1 physically interacts with HY5, which marks it for degradation via the 26S proteasome pathway [28,29].

Under natural light conditions, the overexpression and loss of OsCSN2 have significant effects on rice plant height during the seedling and tillering stages. *OsCSN2*-*OX* with lysine point mutants exhibit distinct phenotypic traits, including larger leaf angles, narrower leaves, deeper green coloration, a dwarf phenotype, and increased tillering [30]. However, under red and far-red light, OsCSN2 overexpressing and knockout plants display traits that differ from those observed under natural light. We analyzed the phenotypes, endogenous hormone levels, and the expression of related genes and proteins in OsCSN2-overexpressing and knockout seedlings under red and far-red light to explore the signaling pathways through which OsCSN2 regulates seedling traits in rice under these light conditions.

## 2. Results

### 2.1. Impact of Lysine Point Mutation in OsCSN2 on Its Interaction with OsCULs

**Figure 1:**
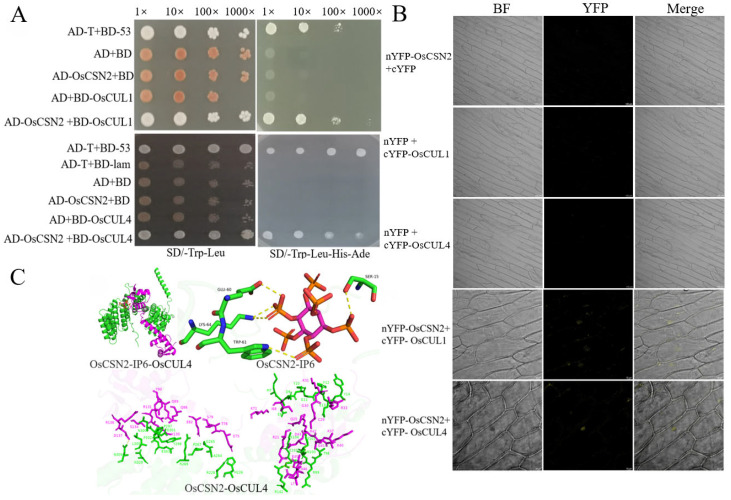
The lysine point mutation in OsCSN2 influences its interaction with OsCULs. (**A**) Yeast two-hybrid assay confirming interactions between OsCSN2 and OsCUL1, and OsCSN2 and OsCUL4; (**B**) BiFC assay verifying interactions between OsCSN2 and OsCUL1, and OsCSN2 and OsCUL4; (**C**) Molecular docking model of OsCSN2 with IP6 and OsCUL4, green represents OsCSN2, and pink represents OsCUL4.

CSN interacts with Cullin through CSN2, participating in Cullin-mediated ubiquitination reactions. In human cells, the binding site of CSN2 with Cullin has been characterized using cryo-electron microscopy [4,31]. However, there have been no related reports in rice. Co-transformation of prey vector OsCSN2 and bait vectors BD-OsCUL1 and BD-OsCUL4 into AH109 yeast competent cells was performed. On SD/-Trp/-Leu plates, all yeast strains grew normally. Subsequently, positive yeast clones were serially diluted and spotted onto SD/-Ade/-Trp/-Leu/-His/3-AT plates. Apart from the positive control (pGADT7-T + pGBKT7-53), OsCSN2 was found to interact with OsCUL1 and OsCUL4 (Figure 1A). The interaction was further validated by Bimolecular Fluorescence Complementation (BiFC) assays. Construct pairs nYFP-OsCSN2/cYFP-OsCUL1 and nYFP-OsCSN2/cYFP-OsCUL4 were co-transformed into *Agrobacterium tumefaciens* EHA105 competent cells. After infiltration into the inner epidermal cells of red onion, significant yellow fluorescence was observed under a laser confocal microscope for both nYFP-OsCSN2 + cYFP-OsCUL1 and nYFP-OsCSN2 + cYFP-OsCUL4 treatments. In contrast, no yellow fluorescence was detected in any other treatment groups (Figure 1B).

Moreover, using molecular docking, the binding sites of OsCSN2 on IP6 and OsCUL4 were predicted. IP6 was found to interact with multiple amino acid residues in OsCSN2, with E60, S15, K64, and W61 forming hydrogen bonds with IP6. Additionally, multiple interaction sites were identified between OsCSN2 and OsCUL4, including the interaction between OsCSN2K104 and OsCUL4 D43 (Figure 1C). The predicted binding sites suggest that the interaction between OsCSN2 and Cullin in rice may be dependent on the involvement of IP6. The lysine residue in CSN2 is a potential critical site for its interaction with Cullin. However, further validation is required to confirm this hypothesis.

### 2.2. OsCSN2 Regulates the Growth of the Rice Stem and Coleoptile Under Red and Far-Red Light Conditions

**Figure 2:**
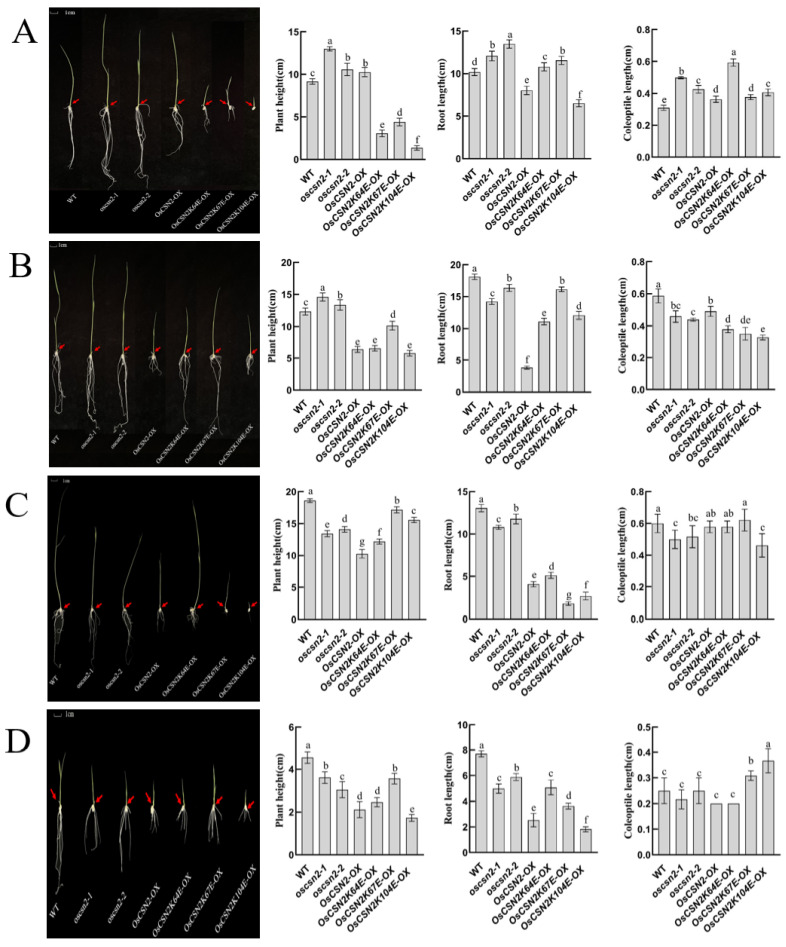
Phenotypic analysis, plant height, root length, and coleoptile length of *oscsn2* under natural light and red light conditions. (**A**) Natural light treatment; (**B**) Red light treatment; (**C**) Red light and GA3 combined treatment; (**D**) Red light and PAC combined treatment. The red arrow indicates the position of the coleoptile. Lowercase letters indicate significant differences (*p* < 0.05).

Under natural light, compared with the WT, *oscsn2* exhibited a significant increase in plant height, with *oscsn2* being taller than the *OsCSN2*-*OX*. In contrast, point mutants showed a significant reduction in plant height, with *OsCSN2K104E*-*OX* displaying the shortest stature. The root length of *oscsn2* was significantly increased, whereas *OsCSN2*-*OX* exhibited markedly reduced root length. *OsCSN2K104E*-*OX* roots were significantly shorter than *OsCSN2*-*OX*, while the other point mutants showed significantly increased root lengths (Figure 2A). The coleoptile length was significantly increased in all mutants, with *OsCSN2K64E*-*OX* exhibiting the longest coleoptile (Figure 2A).

Under red light, the plant height of *OsCSN2*-*OX* was significantly reduced, while the root length of *oscsn2* was significantly shorter. Coleoptile length was significantly reduced in all mutant lines compared with the WT, differing from growth patterns observed under natural light conditions (Figure 2B). Under red light and exogenous GA_3_ treatment, the coleoptile length of *OsCSN2*-*OX*, as well as *OsCSN2K64E*-*OX* and *OsCSN2K67E*-*OX*, increased relative to controls, approaching the WT level (Figure 2C). Under red light and exogenous PAC treatment, no significant difference in coleoptile length was observed between the *oscsn2*, *OsCSN2*-*OX*, *OsCSN2K64E*-*OX*, and WT. However, *OsCSN2K67E*-*OX* and *OsCSN2K104E*-*OX* exhibited significantly longer coleoptiles than the WT (Figure 2D).

**Figure 3:**
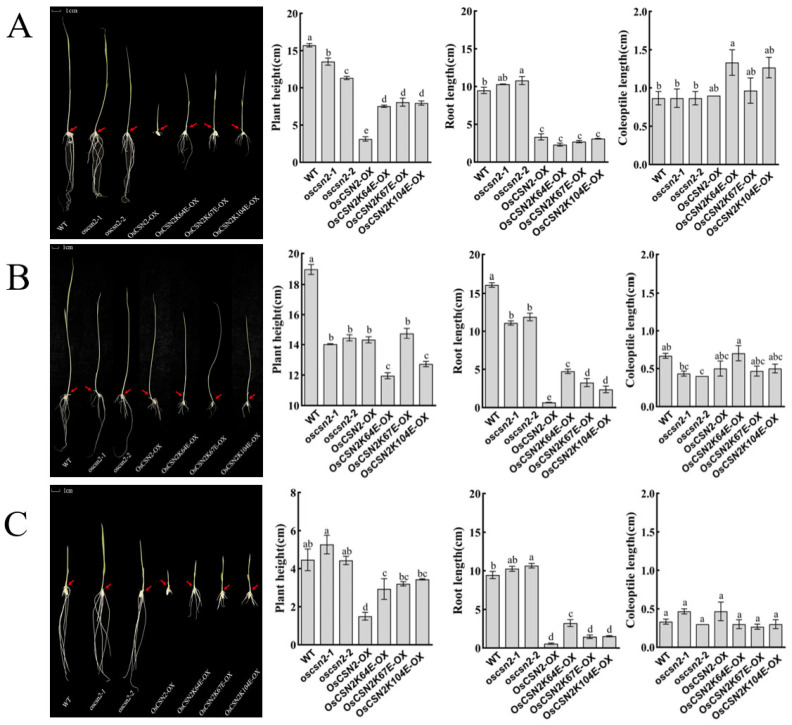
Phenotypic analysis, plant height, root length, and coleoptile length of *oscsn2* under far-red light conditions. (**A**) Far-red light treatment; (**B**) Far-red light and GA_3_ combined treatment; (**C**) Far-red light and PAC combined treatment. The red arrow indicates the position of the coleoptile. Lowercase letters indicate significant differences (*p* < 0.05).

Under far-red light, all *oscsn2* showed a significant reduction in plant height compared to the WT, with the plant height of point mutants being significantly higher than that of *OsCSN2*-*OX* (Figure 3A). Compared to WT, the root length of *oscsn2* increased, while the root length of *OsCSN2*-*OX* and point mutants was significantly reduced, with no significant difference in root length between *OsCSN2*-*OX* and point mutants. The WT exhibited the shortest coleoptile length, and only *OsCSN2K64E*-*OX* showed a significantly longer coleoptile than the WT. Under far-red light and exogenous GA_3_ treatment, coleoptile length in *oscsn2* was shorter than in the WT, whereas *OsCSN2K64E*-*OX* plants exhibited significantly longer coleoptiles (Figure 3B). Under far-red light and exogenous PAC treatment, no significant differences in coleoptile length were observed among all plant lines (Figure 3C).

Under natural light, *OsCSN2-OX* has a greater plant height than the WT. In previous records, plants at the tillering stage also exhibited the same characteristic [30], which may be due to *OsCSN2-OX* affecting the overall function of the CSN complex, thereby indirectly promoting stem growth. The significant reduction in plant height in point mutants may be attributed to the lysine mutation in OsCSN2, which weakens its interaction with Cullin, leading to a decrease in SCF and CRL-mediated substrate degradation, thereby inhibiting stem elongation.

However, under red light, there was no significant change in plant height in *oscsn2*. In contrast, *OsCSN2*-*OX* exhibited a marked reduction in height, and plant height in point mutants also changed. Furthermore, exogenous GA_3_ treatment significantly increased the height of point mutants, while exogenous PAC treatment also affected the height changes in *OsCSN2*-*OX* mutants. This suggests that red light-mediated regulation of rice plant height is associated with GA_3_ signaling.

Under natural light, the regulation of root length by OsCSN2 is similar to its regulation of plant height in rice. However, point mutants exhibited inconsistent root lengths, suggesting that OsCSN2 regulates plant height and root development via distinct functional sites. Under both red and far-red light, the exogenous application of GA_3_ and PAC did not significantly affect the root length of the mutant plants.

Under natural light, coleoptile length was greater in all mutant plants than in the WT. In contrast, under red light, coleoptile length was reduced in all mutant plants compared with the WT. Exogenous GA_3_ application increased the relative length of the coleoptile in mutant plants. In contrast, exogenous PAC treatment resulted in a phenotype opposite to that observed with red light and GA_3_ combined treatment, suggesting that red light regulates coleoptile growth in rice by affecting the accumulation of GA_3_. Under far-red light, neither far-red light alone nor the combination of far-red light with GA_3_ or PAC had a significant impact on the coleoptile phenotype compared to controls, indicating that the effect of far-red light on coleoptile growth is independent of GA_3_ signaling.

### 2.3. OsCSN2 Regulates the Accumulation of GA_3_ in Rice Under Red and Far-Red Light Conditions

**Figure 4:**
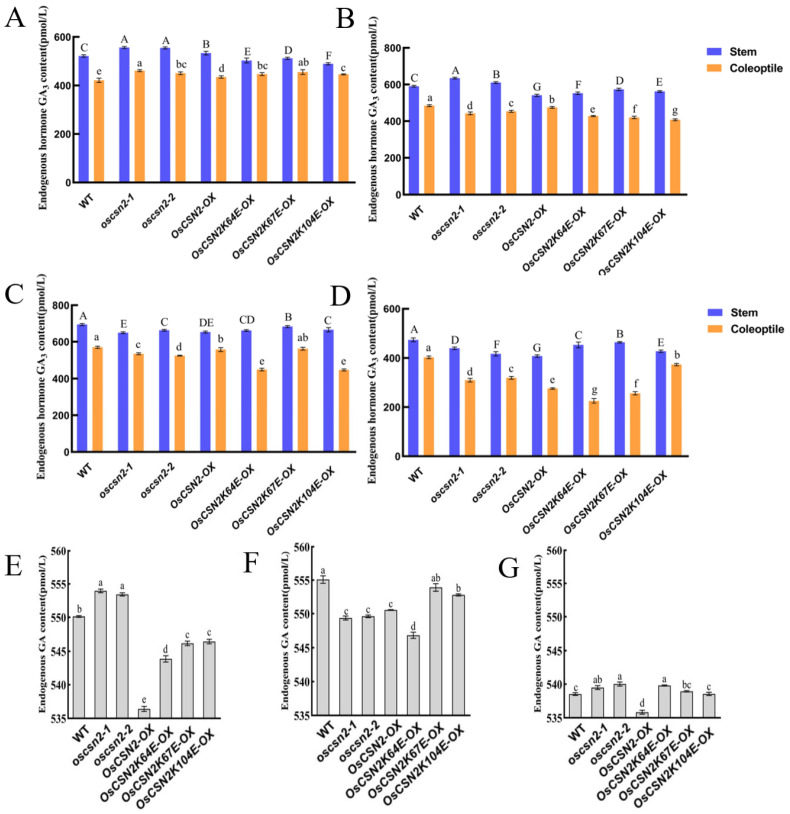
Analysis of endogenous GA_3_ content in *oscsn2* under red and far-red light conditions. (**A**) Natural light treatment; (**B**) Red light treatment; (**C**) Red light and GA_3_ combined treatment; (**D**) Red light and PAC combined treatment; (**E**) Far-red light treatment; (**F**) Far-red light and GA_3_ combined treatment; (**G**) Far-red light and PAC combined treatment. In (**A**–**D**), uppercase letters indicate significant differences in GA3 content in rice stems (*p* < 0.05); lowercase letters indicate significant differences in GA3 content in rice coleoptiles (*p* < 0.05); in (**E**–**G**), lowercase letters indicate significant differences in GA3 content in rice stems (*p* < 0.05).

Under natural light, endogenous GA_3_ levels in the stems of *oscsn2* and *OsCSN2-OX* were significantly higher than in the WT. In contrast, the endogenous GA_3_ content in the stems of point mutants was significantly lower (Figure 4A). Endogenous GA_3_ levels in the coleoptiles of all mutant lines were significantly higher than in the WT, with the *oscsn2-1* exhibiting the highest GA_3_ content.

Under red light, the endogenous GA_3_ content in the stems of *oscsn2* was significantly higher compared to the WT. In contrast, the endogenous GA_3_ content in the stems of *OsCSN2*-*OX* and point mutants was significantly lower. The endogenous GA_3_ content in the coleoptiles of all mutant lines was significantly lower than in the WT (Figure 4B). Under red light and exogenous GA_3_ treatment, the endogenous GA_3_ content in the coleoptile of Os*CSN2K67E*-*OX* did not differ significantly from that in the WT. At the same time, all other mutant lines showed significantly lower GA_3_ levels in the coleoptile (Figure 4C). Under red light and exogenous PAC treatment, endogenous GA_3_ levels in the coleoptiles of all mutant plants were significantly lower than in the WT (Figure 4D).

Under far-red light, the endogenous GA_3_ content in *oscsn2* was significantly higher than that of the WT. In contrast, the GA_3_ content in *OsCSN2*-*OX* and point mutants was significantly lower than that of WT (Figure 4E). Under far-red light and GA_3_ treatment, the endogenous GA_3_ content in all mutant plants was lower than in the WT (Figure 4F). Under far-red light and PAC treatment, the endogenous GA_3_ content in *oscsn2* and *OsCSN2K64E*-*OX* plants was significantly higher relative to the WT (Figure 4G).

Analysis of endogenous GA_3_ content in rice indicates that, unlike treatment with natural light, OsCSN2 has different effects on GA_3_ accumulation in stems and coleoptiles under red light, with GA_3_ content in stems being more sensitive to GA signaling. Under far-red light, OsCSN2 negatively regulates GA_3_ accumulation in stems, and the endogenous GA_3_ content in the stems of all plants can be suppressed by exogenous PAC treatment. However, exogenous GA_3_ treatment leads to complex phenotypes, suggesting that the regulatory process of OsCSN2 on stem GA_3_ accumulation under far-red light is relatively complex.

### 2.4. OsCSN2 Regulates the Expression of Genes Involved in GA Signaling Pathways Under Red Light

**Figure 5:**
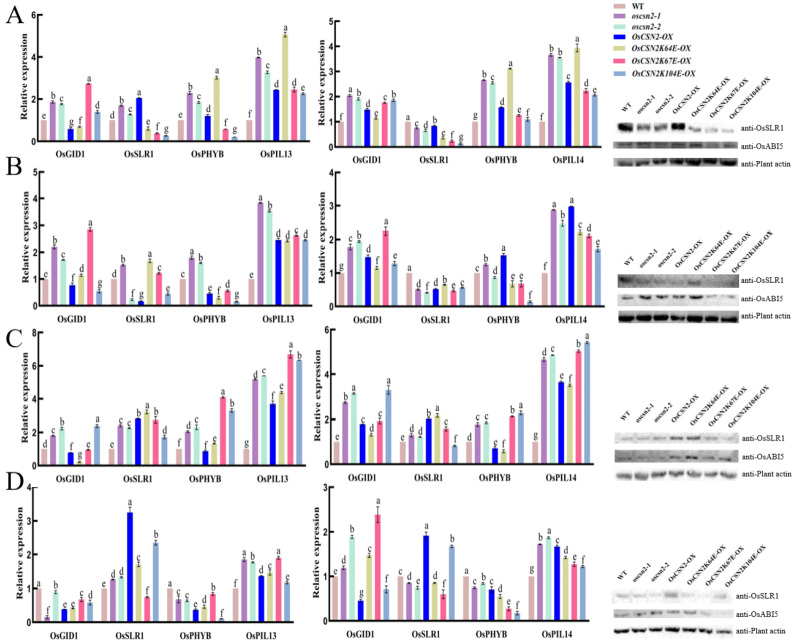
Relative expression analysis of GA signaling pathway genes in the stems and coleoptiles of *Oscsn2*, and protein expression in the stems under natural light and red light conditions. (**A**) Natural light treatment; (**B**) Red light treatment; (**C**) Red light and GA_3_ combined treatment; (**D**) Red light and PAC combined treatment. Lowercase letters indicate significant differences (*p* < 0.05).

To investigate the role of *OsCSN2* in integrating GA and light signaling, we analyzed the expression of key GA pathway genes (*OsGID1* and *OsSLR1*) and photomorphogenesis-related genes (*OsPHYB*, *OsPIL13*, and *OsPIL14*) in the stems and coleoptiles of *oscsn2*, overexpression lines, and lysine point-mutant overexpression plants under red light and natural light conditions (Figure 5).

Under red light, *OsCSN2*-associated transcriptional regulation was particularly pronounced, indicating a central role for *OsCSN2* in red-light signaling. *OsPIL13* and *OsPIL14* were significantly upregulated in all mutant and overexpression lines compared with WT, suggesting enhanced activation of the PHYB–PIL signaling module. In stems, *oscsn2* exhibited elevated expression of *OsGID1* and *OsPHYB*, whereas *OsCSN2-OX* showed reduced expression of *OsGID1*, *OsSLR1*, and *OsPHYB*. In contrast, lysine point-mutant overexpression lines displayed strong suppression of *OsPHYB* expression. Among these lines, *OsCSN2K67E-OX* showed the highest *OsGID1* expression, while *OsCSN2K64E-OX* exhibited relatively higher *OsSLR1* expression, indicating functional divergence of conserved lysine residues in OsCSN2 under red light.

In coleoptiles exposed to red light, *OsSLR1* expression was consistently reduced across all mutant backgrounds, whereas *OsGID1* and *OsPHYB* were markedly induced in *OsCSN2-OX*. Protein analyses further supported these findings, revealing reduced OsSLR1 accumulation in all mutant lines, with the lowest levels detected in *OsCSN2K67E-OX*. OsABI5 protein levels were elevated in OsCSN2 knockout and overexpression plants but were markedly reduced in *OsCSN2K67E-OX*, falling below WT levels.

In contrast, transcriptional regulation under natural light was generally less polarized and served as a baseline for red-light-specific effects. In stems, *oscsn2* exhibited increased expression of all examined genes relative to WT, whereas *OsCSN2-OX* showed reduced *OsGID1* expression but increased OsSLR1, *OsPHYB,* and *OsPIL13* levels. Lysine point-mutant overexpression lines again displayed distinct expression patterns, with *OsCSN2K67E-OX* showing the highest *OsGID1* expression and *OsCSN2K64E-OX* exhibiting the strongest induction of O*sPHYB* and *OsPIL13*. In coleoptiles under natural light, *OsGID1*, *OsPHYB*, and *OsPIL14* were uniformly upregulated across mutant lines, while OsSLR1 expression remained consistently reduced.

Additional treatments combining red light with GA_3_ or PAC further modulated GA- and light-responsive gene expression; however, these effects were comparatively moderate, indicating that OsCSN2-mediated regulation of GA–light crosstalk is primarily established under red light conditions.

### 2.5. OsCSN2 Regulates the Expression of Photomorphogenesis Pathway Genes Under Far-Red Light Conditions

**Figure 6:**
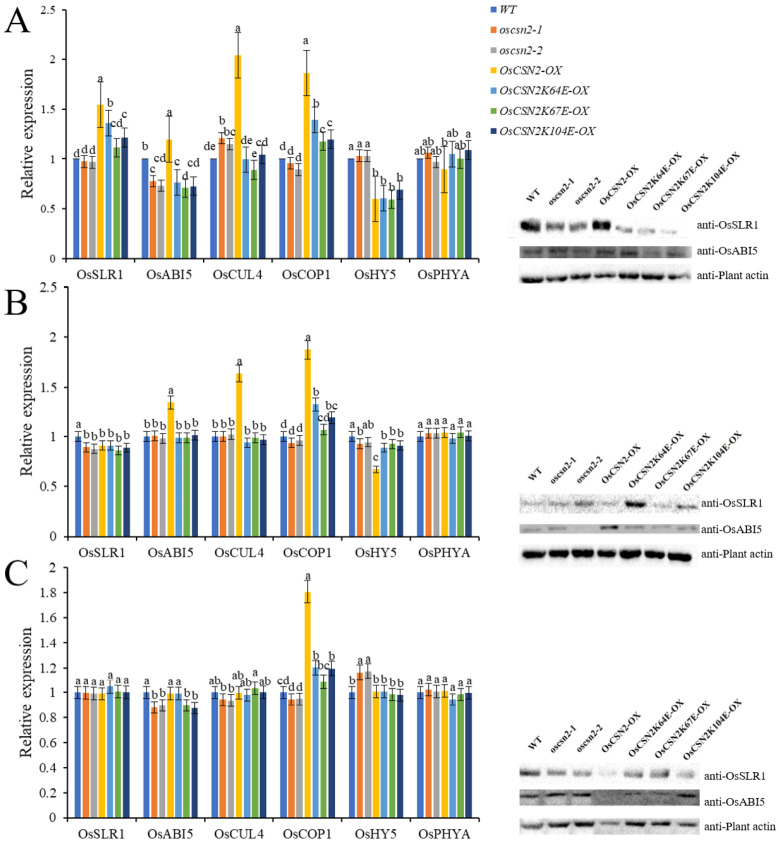
Relative expression analysis of GA signaling pathway genes and proteins in the stems of o*scsn2* under far-red light conditions. (**A**) Far-red light treatment; (**B**) Far-red light and GA_3_ combined treatment; (**C**) Far-red light and PAC combined treatment. Lowercase letters indicate significant differences (*p* < 0.05).

Under far-red light, the expression of *OsSLR1*, *OsCUL4*, *OsPHYA*, *OsCOP1*, *OsHY5*, and *OsABI5* were systematically analyzed across different genetic backgrounds. Compared with the WT, oscsn2 exhibited a significant increase in *OsCUL4* expression and a concomitant decrease in *OsABI5* expression. In contrast, *OsCSN2-OX* under far-red light showed significantly elevated expression of *OsSLR1*, *OsABI5*, *OsCUL4*, and *OsCOP1*, accompanied by reduced *OsHY5* expression, indicating that *OsCSN2* positively regulates far-red light–mediated photomorphogenic signaling (Figure 6).

Consistent with this observation, all point mutants displayed significantly lower expression levels of *OsSLR1*, *OsABI5*, *OsCUL4*, and *OsCOP1* than *OsCSN2-OX* under far-red light. Notably, *OsCSN2K104E*-*OX* exhibited markedly increased *OsPHYA* expression, suggesting that this residue may contribute to the fine regulation of far-red light signaling.

Under far-red light combined with hormonal treatments, the transcriptional responses largely reflected the dominant effect of far-red light. Upon far-red + GA_3_ treatment, OsSLR1 expression was significantly reduced in oscsn2, whereas *OsABI5*, *OsCUL4*, and *OsCOP1* remained highly expressed in *OsCSN2-OX*. Under far-red + PAC treatment, oscsn2 showed elevated *OsHY5* expression, while *OsCSN2-OX* exhibited increased *OsCOP1* expression. Collectively, these results demonstrate that *OsCSN2* plays a central role in integrating far-red light and hormone signaling during photomorphogenesis.

## 3. Discussion

### 3.1. OsCSN2 Regulates Rice Growth by Modulating GA Homeostasis via SLR1 Expression and Controlling Photomorphogenesis Through the COP1–HY5 Complex

GA is a diterpenoid phytohormone that regulates plant growth and development throughout the entire plant life cycle. Among the various GA forms, GA_3_ represents the major bioactive component, controlling multiple developmental processes, including seed germination, stem and hypocotyl elongation, and flowering [32]. In rice, OsCSN2 plays a critical role in maintaining GA homeostasis through several interconnected regulatory pathways.

**Figure 7:**
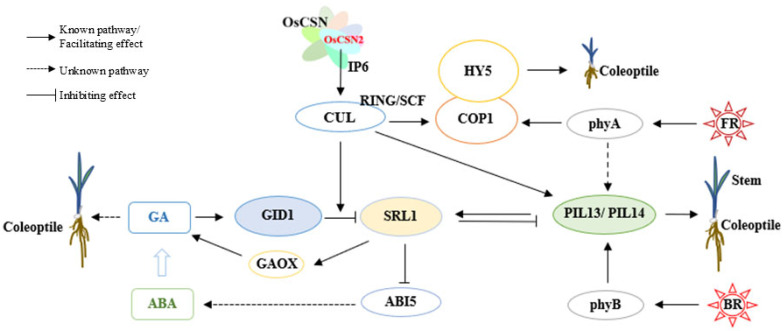
Mechanisms of OsCSN2-Mediated Regulation of Stem and Coleoptile Growth in Rice Through Interaction with OsCULs Under Red and Far-Red Light.

Specifically, OsCSN2 regulates GA dynamics through three principal mechanisms. First, OsCSN2 interacts with OsCULs to modulate GID1-mediated ubiquitination and degradation of the DELLA repressor OsSLR1, thereby directly affecting GA signaling and accumulation. Second, OsCSN2 influences OsSLR1 expression, which in turn regulates the transcription of GA biosynthetic genes such as GAOX, leading to changes in endogenous GA levels. Third, OsCSN2 modulates OsABI5 expression via OsSLR1, thereby affecting ABA accumulation; this ABA-dependent feedback mechanism further contributes to the fine-tuning of GA homeostasis. Among these regulatory routes, OsCSN2-mediated control of GID1-dependent OsSLR1 degradation constitutes the most direct and predominant mechanism governing GA dynamics in rice (Figure 7).

Under low GA conditions, OsSLR1 accumulates and promotes the degradation of PIFs through CRL complexes, thereby facilitating hypocotyl elongation. In addition, OsSLR1 can directly interact with PIF proteins, suppressing their transcriptional activity and rendering PIFs functionally inactive [33,34]. By contrast, when GA levels increase, GID1 binds OsSLR1 to form the GID1–GA–SLR1 complex, which triggers rapid ubiquitination and proteasome-mediated degradation of OsSLR1. The removal of OsSLR1 subsequently releases PIF activity, resulting in the inhibition of hypocotyl elongation.

Under natural light conditions, loss of OsCSN2 function reduces its association with CRL complexes, thereby enhancing GID1-mediated degradation of OsSLR1. This process leads to increased GA_3_ accumulation in rice stems and consequently promotes stem elongation. In contrast, overexpression of OsCSN2 strengthens its interaction with CRLs, suppresses GID1-dependent OsSLR1 degradation, and indirectly elevates GA_3_ levels by upregulating GAOX and repressing OsABI5 expression. Notably, the indirect promotion of GA_3_ accumulation in OsCSN2 overexpression lines is weaker than the effect observed in *oscsn2* loss-of-function mutants. This difference provides a mechanistic explanation for why both *OsCSN2-OX* and *oscsn2* exhibit increased plant height, with *oscsn2* showing a more pronounced elongation phenotype (Figure 7).

Light signaling further integrates GA regulation with photomorphogenic control. Upon light perception, phytochromes promote photomorphogenesis primarily by inhibiting COP1-mediated degradation of the transcription factor HY5, through two major mechanisms. One mechanism involves light-induced changes in COP1 subcellular localization, resulting in its translocation from the nucleus to the cytoplasm [35]. The other mechanism entails direct interactions between photoactivated phytochromes and COP1 or SPA1, which disrupt the formation of the COP1/SPA1 E3 ubiquitin ligase complex, thereby allowing the nuclear accumulation of HY5 and other positive regulators of photomorphogenesis [36,37]. Under natural light conditions, loss of OsCSN2 reduces its association with CRLs, promotes the formation of the COP1/SPA1 E3 ubiquitin ligase complex, and enhances OsHY5 accumulation, ultimately facilitating hypocotyl elongation in rice.

### 3.2. OsCSN2 Lysine Single-Point Mutations Regulate Rice Growth by Modulating the Interaction Between the CSN Complex and CRLs

The CSN regulates E3 ubiquitin ligase activity through deneddylation, thereby controlling the ubiquitin–proteasome system and protein degradation. This function depends on the interaction between CSN and CULs [38]. Yeast two-hybrid and BiFC assays demonstrated that OsCSN2 interacts directly with OsCUL1 and OsCUL4, indicating that the OsCSN complex associates with CRLs via OsCSN2 to participate in CRL-mediated ubiquitination. Lysine residues in OsCSN2 constitute the primary sites for OsCUL binding, and ubiquitination site prediction identified K64, K67, K71, and K104 as high-probability ubiquitination sites.

IP6 markedly enhances CSN–CRL4 association, and its binding involves seven lysine residues in CSN2 [11,12]. Molecular docking analysis predicted that IP6 interacts with multiple OsCSN2 residues, with OsCSN2K64 forming hydrogen bonds with IP6 and OsCSN2K104 interacting with OsCUL4D43. Functional analysis of single-point mutation overexpression lines revealed that, under natural light conditions, *OsCSN2K64E-OX* exhibits the highest expression of OsphyB, OsPIL13, and OsPIL14 in stems and coleoptiles, together with the longest coleoptile length. Mutation of lysine 64 reduces IP6 binding sites, impairs OsCSN2 recognition of unneddylated CRLs, and enhances CRL-mediated ubiquitination and degradation of OsPIL13 and OsPIL14.

In Arabidopsis, CRLs involved in substrate ubiquitination are mainly composed of CUL1-, CUL3-, and CUL4-based complexes [39]. Although these CRLs differ in substrate specificity, all three participate in light-induced degradation of PIFs [40]. Phytochrome-dependent regulation of PIF activity modulates phytohormone biosynthesis and signaling, thereby directly affecting the expression of genes controlling stem growth [41]. As key components of phytochrome signaling, PIFs are phosphorylated by activated phytochromes and subsequently ubiquitinated by CRLs, playing a central role in photomorphogenesis [42].

In rice, OsPIL13 functions as a transcription factor that negatively regulates phyB-dependent light signaling while positively regulating internode elongation [43]. OsPIL14 preferentially interacts with the photoactivated form of OsphyB, showing stronger binding to the C-terminal domain of OsphyB than to OsphyA, which leads to sequestration or targeted degradation of PIL14 [44]. Accordingly, lysine mutations in OsCSN2 disrupt its interaction with CRLs, accelerate the ubiquitination and degradation of OsPIL13 and OsPIL14, and ultimately modulate rice stem elongation and coleoptile growth.

### 3.3. Under Red Light, OsCSN2 Regulates GA Homeostasis and Rice Growth by Modulating SLR1 Degradation via Interaction with CULs

Under red light conditions, OsCSN2 displays a regulatory mode distinct from that under natural light. Both *oscsn2* and *OsCSN2-OX* exhibit significantly reduced coleoptile length, whereas only *OsCSN2-OX* shows a marked decrease in plant height, indicating that OsCSN2 negatively regulates plant height under red light. Notably, *OsCSN2K67E*-*OX* plants are significantly taller than *OsCSN2*-OX, suggesting a critical role for Lys67 in this regulation.

Red light strongly suppresses primary root and coleoptile growth in all mutant lines. Compared with *OsCSN2*-*OX*, single-point mutation overexpression lines—especially K67E—partially relieve root growth inhibition while enhancing repression of coleoptile elongation, indicating that key amino acid substitutions alter OsCSN2 organ-specific regulatory bias [9].

At the molecular level, phyB activation under red light induces PIL13 and PIL14 expression in all genotypes. Concurrently, GA_3_ accumulation and OsSLR1 expression are reduced in *OsCSN2*-*OX* stems and coleoptiles, whereas single-point mutation overexpression lines show elevated OsSLR1 expression and increased GA_3_ levels. Consistently, OsGID1 expression is highest in *OsCSN2K67E*-*OX* and overall higher in *oscsn2* than in *OsCSN2-OX*, correlating with their growth phenotypes.

Collectively, these results indicate that red light shifts OsCSN2 function from a PIF-centered regulatory network to SLR1-dependent control of GA homeostasis. Mutation of Lys67 weakens OsCSN2–CULLIN interaction, accelerates CRL-mediated OsSLR1 degradation, enhances GA_3_ accumulation, and promotes formation of the GID1–GA–SLR1 complex, thereby reprogramming rice growth responses specifically under red light [45].

### 3.4. OsCSN2 Lysine Single-Point Mutations Specifically Promote Rice Coleoptile Elongation Under Far-Red Light

Under far-red light conditions, plant height is significantly reduced in all mutant genotypes, indicating that far-red light broadly suppresses shoot elongation in rice. In contrast, root and coleoptile growth display clear genotype-dependent differences. The primary roots of *oscsn2* are significantly elongated, whereas OsCSN2 overexpression and single-point mutation overexpression lines exhibit markedly shorter primary roots than the wild type. Conversely, single-point mutation overexpression lines develop significantly longer coleoptiles than the wild type, *oscsn2*, and *OsCSN2*-*OX*, suggesting that mutations at key residues of OsCSN2 specifically promote coleoptile elongation under far-red light.

At the transcriptional level, *OsCSN2*-*OX* shows the highest expression of OsCUL4 and OsCOP1 under far-red light, while single-point mutation overexpression lines exhibit significantly higher OsCOP1 expression than *oscsn2* and the wild type. Despite these differences, OsHY5 expression does not differ significantly between *OsCSN2*-*OX* and single-point mutation overexpression plants and remains consistently lower than in *oscsn2* and the wild type, indicating that COP1-mediated repression of photomorphogenesis predominates under far-red light.

Mechanistically, single-point mutations in OsCSN2 reduce its binding interfaces with IP6 and OsCUL4, thereby weakening the ability of theCSN to recognize and associate with unneddylated CRLs. Because the CUL4–COP1/SPA complex is a key E3 ubiquitin ligase in phytochrome-mediated light signaling [46], these mutations reduce competition between CSN and COP1 for CUL4 binding, shifting the equilibrium between the inactive CRL4–CSN complex and the active CRL4–COP1/SPA E3 ligase complex [47,48].

This shift in complex assembly under far-red light enhances CRL4–COP1/SPA activity and reprograms photomorphogenic signaling, ultimately resulting in pronounced coleoptile elongation in single-point mutation overexpression lines. Collectively, these results demonstrate that OsCSN2 modulates organ-specific growth responses under far-red light by fine-tuning the dynamic balance of CSN–CRL4–COP1/SPA complexes.

## 4. Materials and Methods

### 4.1. Prediction of OsCSN2 Ubiquitination Sites and Mutant Development

OsCSN2 (LOC4326902) has a full length of 7057 bp, comprising 12 exons, and encodes a CDS of 1320 bp, which in turn produces a protein of 439 amino acids. The potential lysine ubiquitination sites in OsCSN2 were predicted using the iRice-MS platform (http://lin-group.cn/server/iRice-MS/webServer.html). Lysine residues at positions 58, 64, 67, 71, 75, 78, 80, and 428 had a high probability (>90%) of undergoing ubiquitination, while lysines at positions 34, 94, 104, 239, 259, 416, and 422 had an 80–90% probability of ubiquitination.

In previous experiments, *oscsn2* were generated, and on a homozygous *OsCSN2* knockout background, we constructed complemented lines, including *OsCSN2*-*OX*, *OsCSN2K64E*-*OX*, *OsCSN2K67E*-*OX*, and *OsCSN2K104E*-*OX*. Both *oscsn2* and *OsCSN2*-*OX* mutants displayed significant increases in plant height. However, the *OsCSN2K71E*-*OX* mutant exhibited a high lethality rate and very low fertility and was therefore excluded from further studies [30]. The WT rice used in this study was *Oryza sativa* L. spp. *japonica*. The materials used in this study, including *oscsn2* mutants (*oscsn2-1* and *oscsn2-2*), *OsCSN2* Overexpression mutants (*OsCSN2*-*OX*), and lysine point mutants (*OsCSN2K64E*-*OX*, *OsCSN2K67E*-*OX*, and *OsCSN2K104E*-*OX*), were maintained and propagated in our laboratory. The construction and transformation methods have been detailed in a previously published study.

### 4.2. Molecular Docking Prediction of OsCSN2 and OsCUL4

Protein sequences of OsCSN2 and OsCUL4 were downloaded, and their structures were retrieved from the AlphaFold Protein Structure Database (AlphaFold DB, https://alphafold.ebi.ac.uk (accessed on 8 June 2024)). Hydrogen atoms were removed from the structures using PyMOL 2.5. Protein docking of OsCSN2 and OsCUL4 was performed using MOE 2019, whereas IP6 was optimized using the MMFF94 force field [38]. Docking between OsCSN2 and IP6 was conducted using AutoDock Vina (https://vina.scripps.edu/ (accessed on 8 June 2024)) with molecular docking centered at the coordinates x = 15.751, y = 11.443, and z = −32.434. A genetic algorithm was applied for conformational sampling and scoring, and the optimal conformation was selected based on docking scores. Binding mode analysis and visualization were conducted using MOE 2019 and PyMOL (https://www.pymol.org/ (accessed on 8 June 2024)).

### 4.3. Yeast Two-Hybrid and Bimolecular Fluorescence Complementation Analysis

In a yeast two-hybrid experiment, OsCSN2, OsCUL1, and OsCUL4 were cloned. The vectors pGADT7 and pGBKT7 were double-digested with the restriction enzymes EcoRI and BamHI and ligated with recombinase to construct the recombinant plasmids pGADT7-OsCSN2, pGBKT7-OsCUL1, and pGBKT7-OsCUL4. The experimental groups were pGADT7-OsCSN2 + pGBKT7-OsCUL1 and pGADT7-OsCSN2 + pGBKT7-OsCUL4. The positive control was pGADT7-T + pGBKT7-53, whereas the negative controls included pGADT7-T + pGBKT7-Lam, pGADT7 + pGBKT7-OsCUL1/OsCUL4, and pGADT7-OsCSN2 + pGBKT7. Plasmids from experimental and control groups were extracted and transformed into AH109 yeast-competent cells. The cultures were incubated at 30 °C for three days on SD/-Trp/-Leu and SD/-Ade/-Trp/-Leu/-His/3-AT plates. The primer sequences used are listed in Table 1.

In the BiFC assay, the full-length CDS of *OsCSN2*, *OsCUL1*, and *OsCUL4* were cloned into pX-nYFP/pX-cYFP vectors under the control of the 35S promoter. *Agrobacterium* strains carrying each construct were co-infiltrated into the inner epidermis of the purple onions. After 48 h, fluorescence in the epidermal cells was examined using a confocal laser scanning microscope. Primer sequences are provided in Table 1.

### 4.4. Red and Far-Red Light Treatments and Phenotypic Analysis

Seeds from the WT, *oscsn2-1*, *oscsn2-2*, *OsCSN2-OX*, *OsCSN2K64E-OX*, *OsCSN2K67E-OX*, and *OsCSN2K104E-OX* lines were surface-sterilized and sown on 0.65% agar medium. One group was designated as the control and placed in an incubator set to a 12 h light/12 h dark cycle at 28 °C for 9 days. Three additional groups were transferred onto blank solid medium, solid medium supplemented with 5 μM GA_3_, and solid medium with 10 μM PAC and incubated under red light at 28 °C for 9 days. Similarly, the three groups were cultured on blank solid medium, GA_3_-supplemented medium, and PAC-supplemented medium under far-red light at 28 °C for 9 days. After 9 days, phenotypes were documented using photographs, and measurements of plant height, coleoptile length, and primary root length were recorded. The light sources utilized in this study include white light, red light, and far-red light, all of which are LED tubes. The red light exhibits a peak wavelength of 630 nm, while the far-red light has a peak wavelength of 735 nm. All treatments maintain consistent light intensity; the red and far-red light treatments provide continuous illumination for 24 h, whereas the natural light treatment adheres to a 12 h light/12 h dark cycle.

### 4.5. Quantification of Endogenous GA_3_ Levels

Endogenous GA_3_ levels in rice were measured using a GA_3_ ELISA kit (Solarbio, Beijing, China). Stems and roots from 9-day-old wild-type and mutant seedlings were ground thoroughly in 80% pre-cooled methanol. The homogenate was centrifuged at 10,000× *g* for 15 min at 4 °C, and the supernatant was filtered through a Sep-Pak Plus C18 column. The filtrate was vacuum-dried, and the resulting residue was dissolved in methanol for GA_3_ analysis. The detailed procedures were performed according to the ELISA kit instructions.

### 4.6. qPCR and Western Blot Analysis

In the qPCR experiment, total RNA was extracted using the Spectrum Plant Total RNA Kit (Sigma-Aldrich, Taufkirchen, Germany). Starting with 5 μg of total RNA, reverse transcription was performed using the StarScript II First-Strand cDNA Synthesis Mix with gDNA Remover (GenStar, Beijing, China). Quantitative PCR was conducted on a StepOne Plus™ Real-Time PCR system (Thermo Fisher Scientific, Applied Biosystems, Beijing, China) using a 2 × RealStar Green Fast Mixture with ROE (GenStar, China). GAPDH (JN848809) served as an internal control for normalizing relative mRNA levels, with data averaged from three replicates. Primer sequences are provided in Table 1.

For the WB experiment, 9-day-old seedlings were ground in liquid nitrogen. Proteins were extracted using a buffer containing 2.5 mM EDTA, 10 mM MgCl_2_, 50 mM Tris-HCl (pH 7.5), 150 mM NaCl, 0.1% NP-40, 1 mM DTT, 1 mM PMSF, and a complete protease inhibitor cocktail (Roche, Beijing, China). After centrifugation for 10 min, the supernatant was collected, and equal amounts of total protein were added to each sample. The samples were then mixed with 5 × SDS-PAGE sample buffer (Genstar, Beijing, China) and boiled for 10 min. For protein separation, 20 μL of each sample was loaded onto an 8% SDS-PAGE gel and electrophoresed at a constant current of 20 mA until bromophenol blue reached the bottom of the gel. Proteins were transferred onto PVDF membranes using the Trans-Blot Turbo™ Transfer System (Bio-Rad, Hercules, CA, USA) at a constant pressure of 2.5 V for 10 min. Membranes were blocked with 4% nonfat milk, incubated with primary antibodies, and visualized using chemiluminescence. Antibodies included rabbit polyclonal antibodies against OsCSN2, OsCSN5, OsABI5, OsSLR1, and OsCUL4, as well as anti-plant actin, goat anti-rabbit IgG (HRP), and goat anti-mouse IgG (HRP) secondary antibodies, all obtained from Wuhan ABclone Biotechnology Co., Ltd. Protein signals, were detected using a Universal Hood III (731 BR 03292, Bio-Rad, CA, USA).

## 5. Conclusions

This study demonstrates that under red and far-red light, OsCSN2 and its lysine single-point mutants mediate the rice light-hormone crosstalk pathway by regulating the transcription and protein expression of key genes in the GA pathway (*OsGID1*, *OsSLR1*) and photomorphogenesis pathway (*OsCOP1*, *OsPHYB*, *OsPIL13/14*). The results provide important insights into the molecular mechanism of OsCSN in responding to monochromatic light stress and offer theoretical references and genetic resources for the creation and improvement of specific rice varieties. Notably, this study has limitations: first, no ubiquitination-related experiments were conducted; second, the effect of OsCSN2 lysine single-point mutations on ubiquitination in rice was not systematically explored; third, the whole-life cycle phenotypic characteristics of OsCSN2 and its single-point mutant rice under red and far-red light remain unobserved. These issues require further systematic investigation.

## Figures and Tables

**Table 1:** Primers used in this study.

Name	Sequence
OsCSN2-F	CGCTGCGGTCTCGGAAACCC
OsCSN2-R	CAGATAGATTCAGTTCAGGACAAAA
OsCUL1-F	GCCATGGAGGCCGAATTC
OsCUL1-R	TGCAGGTCGACGGATCCC
OsCUL4-F	GCCATGGAGGCCGAATTC
OsCUL4-R	TGCAGGTCGACGGATCCC
pGADT7-OsCSN2-F	ATGGAGGCCAGTGAATTCATGCGGACATGGAGGATTACGGGTT
pGADT7-OsCSN2-R	TCGAGCTCGATGGATCCCCCCAACTCTGTTGGACACCGTTTG
pGBKT7-OsCUL4-F	GCCATGGAGGCCGAATTCATGCACAAAAACTAAGCTTC
pGBKT7-OsCUL4-R	TGCAGGTCGACGGATCCCAGCCAGGTAATTGTAGATCT
pGBKT7-OsCUL1-F	GCCATGGAGGCCGAATTCATGCACAAAAACTAAGCTTC
pGBKT7-OsCUL1-R	TGCAGGTCGACGGATCCCAGCCAGGTAATTGTAGATCT
nYFP-OsCSN2-F	GATCTCGAGCTCAAGCTTCGAATTCATGCGGACATGGAGGATTACGGGTT
nYFP-OsCSN2-R	TCGCCCTTGCTCACCATCAGAATTCCCCAACTCTGTTGGACACCGTTTG
cYFP-OsCUL1-F	GATCTCGAGCTCAAGCTTCGAATTCATGGCGACCCACGAGCGGAA
cYFP-OsCUL1-R	GCGAGCTGCACGCTGCCCAGGATCCAGCCAAGTATCTGTACACCAT
cYFP-OsCUL4-F	GATCTCGAGCTCAAGCTTCGAATTCATGCACAAAAACTAAGCTTC
cYFP-OsCUL4-R	GCGAGCTGCACGCTGCCCAGGATCCAGCCAGGTAATTGTAGATCT
qRT-GAPDH-F	AAGCCAGCATCCTATGATCAGATT
qRT-GAPDH-R	CGTAACCCAGAATACCCTTGAGTTT
qRT-OsSLR1-F	CATGCTTTCCGAGCTCAACG
qRT-OsSLR1-R	TGACAGTGGACGAGGTGGAA
qRT-OsGID1-F	GCTCTGCTCTCTGTCCCTCTCTC
qRT-OsGID1-R	CTCACCACCACCTCCTCCTCAG
qRT-OsABI5-F	AGGGAGGAGGGAGGCGATG
qRT-OsABI5-R	CGTCAGCGAATACACCGAACC
qRT-OsCUL1-F	AGGACAGACAGTATCAGGTGGATGC
qRT-OsCUL1-R	TCCGATGGCTTGATTGGGAACTTG
qRT-OsPHYA-F	GATGGTGCTCTGAGTGGAATGC
qRT-OsPHYA-R	ACAGGAGGCGTTGGTGCTATC
qRT-OsCOP1-F	CATCTCAGCCACAAGAGCGACTG
qRT-OsCOP1-R	GGTCTATCGGTGATGCTGTCTTCG
qRT-OsHY5-F	GCAAGGGAGAGAAAGAAGGCATAC
qRT-OsHY5-F	CAGTATCTGTCTGAGCATCTGGTTC
qRT-OsCSN1-F	CGTCGCCTCGCCTCACCTATCTA
qRT-OsCSN1-R	TAAAACCACAGCAAGCAAGGAAT
qRT-OsCSN5-F	GAGCAAGCTGAGGGTCAACT
qRT-OsCSN5-R	GACCATGGACCTHTTCAGCA
qRT-OsPHYB-F	TAAGGGAGTCGGAGCGGAGTTTC
qRT-OsPHYB-R	AGCCCGCCCGCACCGAGGATCCT
qRT-OsPIL13-F	CGGCGGAAGTGCATAACCTGAG
qRT-OsPIL13-R	TCATCCAGAATGCTCGCTTTATCGG
qRT-OsPIL14-F	CCTGCTGCTTCTTCGTCGCAGTATA
qRT-OsPIL14-R	TTTTGCGAAGACTACTAATAATAAC

## Data Availability

Data are contained within the article.
